# Alzheimer's disease gene signature says: beware of brain viral infections

**DOI:** 10.1186/1742-4933-7-16

**Published:** 2010-12-14

**Authors:** Elisa Porcellini, Ilaria Carbone, Manuela Ianni, Federico Licastro

**Affiliations:** 1 Department of Experimental Pathology, School of Medicine, University of Bologna Italy

## Abstract

**Background:**

Recent findings from a genome wide association investigation in a large cohort of patients with Alzheimer's disease (AD) and non demented controls (CTR) showed that a limited set of genes was in a strong association (p > l0^-5^) with the disease.

**Presentation of the hypothesis:**

In this report we suggest that the polymorphism association in 8 of these genes is consistent with a non conventional interpretation of AD etiology.

Nectin-2 (NC-2), apolipoprotein E (APOE), glycoprotein carcinoembryonic antigen related cell adhesion molecule- 16 (CEACAM-16), B-cell lymphoma-3 (Bcl-3), translocase of outer mitochondrial membrane 40 homolog (T0MM-40), complement receptor-1 (CR-l), APOJ or clusterin and C-type lectin domain A family-16 member (CLEC-16A) result in a genetic signature that might affect individual brain susceptibility to infection by herpes virus family during aging, leading to neuronal loss, inflammation and amyloid deposition.

**Implications of the hypothesis:**

We hypothesized that such genetic trait may predispose to AD via complex and diverse mechanisms each contributing to an increase of individual susceptibility to brain viral infections

## Background

The incidence of Alzheimer's disease (AD) is rising sharply and a large fraction of the elderly population will ultimately be affected by the disease. Because of an urgent need for effective preventative and therapeutic measures, extensive research has focused on pathogenetic mechanisms of the disease. However, effective therapy is not already available. AD pathology is characterized by neuronal loss leading to brain atrophy and a decrement of the cerebral metabolism. Major neuropathologic lesions are: (i) synapse and neuron loss; (ii) extracellular amyloid deposits and amyloid plaques, principally composed of amyloid beta (Aβ) peptide; (iii) intraneuronal accumulation of hyperphosphorylated Tau proteins leading to neurofibrillary degeneration; (iv) reactive astrogliosis; (v) brain inflammation. Current views of AD pathogenetic mechanisms describe amyloid deposition and neuritic plaque formation as a central mechanisms leading to neuro-degeneration, cognitive impairment and sporadic AD [1]. Therefore, therapeutic approaches have focused on reducing amyloid load and plaque deposition or clearance of brain amyloid. Other mechanisms may be closely related with the etiology and pathogenesis of sporadic AD.

## Presentation of the hypothesis

Here we discuss recently published genetic data from a genome wide association (GWA) study including several thousand AD European patients and controls (CTR) [2] and showing that a limited number of genes were highly associated (p > l0^-5^) with the disease even after the inclusion of additional data from control population (stage 3 of GWA replication and statistical evaluation by principal component adjustment [2]). The view presented here supports the notion of an infective etiology for sporadic AD. The first set of genes was located in close vicinity of the APOE locus on the chromosome 19 (see Table 1) and consisted of the poliovirus receptor-related 2 or nectin-2 (NC-2), apolipoprotein E (APOE), the translocase of outer mitochondrial membrane 40 homolog (TOMM-40), the glycoprotein carcinoembryonic antigen related cell adhesion molecule-l6 (CEACAM-l 6) and B-cell/lymphoma-3 (Bcl-3) genes. Genes in the second set were located on different chromosomes: APOJ or clusterin on chromosome 8; the complement receptor 1 (CR-1), and C-type lectin domain family 16 member A (CLEC-16A) on chromosome 16. Polymorphic variations in each of these genes were individually associated with AD (P values ranging from l0^-16 ^to 10^-5^). However we argue that the concomitant presence of several polymorphisms of these genes in the same individual might represent a genetic signature of AD. In this report we hypothesized that such a genetic trait may predispose to AD via complex and diverse mechanisms each contributing to an increase of individual susceptibility to brain viral infections. The evidence supporting this new notion are briefly listed below.

**Table 1:** Genes cluster surrounding the APOE gene on human chromosome 19 Region: 45,120K-45,710 K bp

Start	Stop	Symbol	Cyto	Description
45116956	45138792	LOC147710	19	hypothetical LOC147710

45147098	45169429	PVR	19q13.2	poliovirus receptor

45174724	45187627	CEACAM19	19	carcinoembryonic antigen-related cell adhesion molecule 19

45202358	45213986	CEACAM16	19	carcinoembryonic antigen-related cell adhesion molecule 16

45251978	45263301	BCL3	19q13.1-q13.2	B-cell CLL/lymphoma 3

45281126	45303903	CBLC	19q13.2	Cas-Br-M (murine) ecotropic retroviral transforming sequence c

45312338	45324678	BCAM	19q13.2	basal cell adhesion molecule (Lutheran blood group)

45349393	45392485	PVRL2	19q13.2	poliovirus receptor-related 2 (herpesvirus entry mediator B)

45394477	45406946	TOMM40	19q13	translocase of outer mitochondrial membrane 40 homolog (yeast)

45409039	45412650	APOE	19q13.2	apolipoprotein E

45411802	45431701	LOC100129500	19	hypothetical LOC100129500

45417921	45422606	APOC1	19q13.2	apolipoprotein C-I

45430017	45434450	APOC1P1	19q13.2	apolipoprotein C-I pseudogene 1

45445495	45448751	APOC4	19q13.2	apolipoprotein C-IV

45449243	45452818	APOC2	19q13.2	apolipoprotein C-II

45458638	45496599	CLPTM1	19q13.2-q13.3	cleft lip and palate associated transmembrane protein 1

45504712	45541452	RELB	19	v-rel reticuloendotheliosis viral oncogene homolog B

45542298	45574214	SFRS16	19q13.3	splicing factor, arginine/serine-rich 16

45574758	45579688	ZNF296	19	zinc finger protein 296

45582518	45594782	GEMIN7	19	gem (nuclear organelle) associated protein 7

1) NC-2, also known as herpes virus entrance-B (HveB) or poliovirus receptor-relate protein-2 (PVRL-2 or Prr2), is a member of the immunoglobulin superfamily, is expressed in a variety of cell tissues, including neurons, belongs to the cadherin adhesion molecules [3] and mediates the entry of herpes simplex viruses (HSV) [4]. The glycoprotein D (gD) of HSV is the ligand for NC-2 and one of several HSV binding proteins that are essential for fusion to the human target cell and viral entering [4]. Gene variants in the human NC-2 gene might affect individual susceptibility to HSV infection of the brain by influencing virus cell entry and cell-to cell virus spreading.

2) APOE 4 allele is a well established genetic risk factor for AD and it has been also confirmed in the European GWA study [2]. ApoE protein may affect Aβ deposition. However, APOE 4 allele has been also shown to influence: susceptibility to viral infections [5], human immune deficiency virus (HIV) cell entry *in vitro*, HIV disease clinical progression [6], recurrent genital herpes in patients co-infected by HSV-2 and HIV [7] and progression of experimental ocular lesions induced by HSV-1 [8]. Therefore, APOE 4 allele association with AD might also influence the susceptibility to virus entry and spreading into neuronal cells. On the other hand, APOE 4 allele seems to be protective in the case of liver damage caused by HCV [9].

3) TOMM-40 gene codes for a mitochondrial translocase. It is interesting to note that HSV DNAase such as the UL12.5 enzyme destroys the mitochondrial genome [10] by inducing rapid and complete degradation of mitochondrial DNA [11]. Gene variations at TOMM-40 gene might influence DNA digestion and mitochondrial damages induced by HSV DNAase and other less defined virus dependent mechanisms.

4) CEACAM-16 belongs to a family of gene coding for adhesion molecules related to cancer replication, such as the carcinoembryonic antigen (CEA), and has been recently shown to regulate apoptosis in early tumor development by affecting caspase-l/3 activation [12].

5) Bcl-3 is an oncogene and is also involved in cell replication and apoptosis. Apoptosis may act as a primitive immune response and is a potent host defense mechanism. It is known that HSV is able to both induce and suppress apoptosis in infected cells. In particular HSV-1 was shown to inhibit initially induced apoptosis in neuronal cells via a caspase-3 dependent pathway [13]. Moreover, Bcl-2 protein was able to block HSV-1 induced apoptosis in human hepatocytes [14]. Therefore, gene polymorphism in both CECAM-16 and Bcl-3 genes might influence individual susceptibility to apoptosis regulation induced by HSV and favor virus spreading in the central nervous system (CNS).

6) The APOJ, also known as clusterin, is a modulator of complement activation. Complement biosynthesis and activation occurs in neurodegenerative diseases such as AD [15] and the cytolytic activity of complement components is important for virus neutralization. ApoJ is synthesized in the CNS and is present in amyloid plaques [16]. Polymorphism in APOJ gene might influence virus lytic defences by regulation of complement activation.

7) CR1 is a complement receptor which bind different complement components (C3b, C3 d, C2a). Herpes virus family (especially alpha herpes) expresses a member of gC protein family that is able to bind heparan sulphate and the C3b component of the complement system [4]. Genetic variation in CR1 and CR2 receptors might affect individual capacity of virus clearance via C3 activation and C3b binding to the HSV. APOJ and CR1 genes might be illustrated as a synergistic gene cluster and influence brain virus defences such as complement activation, virus lysis and clearance.

8) CLEC-16A gene codes for a C-type lectin domain receptor. Lectin-like receptor, such as mannose receptor, recognizes and binds sugar moieties on pathogen glycoproteins. No data are on record regarding CLEC-16A and HSV or other viruses. However, gene polymorphism in the CLEC-16A gene might influence individual ability to recognize and bind virus glycoproteins.

## Implications the hypothesis

The genetic signature here discussed is suggestive of individual susceptibility to pathogen infection of the brain, particularly HSV and related viruses. Recently, an independent investigation in late-onset sporadic AD from Japan also showed that gene variations near the APOE locus (PVRL-2, APOE 4 allele and APOC1) on chromosome 19, were associated with increased risk for the disease [17]. These independent findings appear to reinforce the new notion that individual brain susceptibility to virus infection and/or reactivation may be one complex genetic trait influencing the risk of neurodegeneration leading to clinical AD in old age. Moreover, evidence from other investigators showing HSV infection in AD brains are on record [18-20]. It is of interest that the concomitant presence of the APOE 4 allele and vertical transmission of HSV-1 has been shown to confer a differential risk of brain infection and AD [21]. Moreover, APOE 4 deficient mice had significantly lower virus load in CNS than APOE 4 transgenic mice [22]. In addition, in transgenic mouse model, APOE4 was shown to be a risk factor for ocular herpes favoring increased HSV-1 intra ocular replication [23].

Reactivation of HSV-1 in the brain was also found in patients with familial AD who showed increased viral DNA and protein expression in cortical neurons [24]. HSV-1 has been also related to Down's syndrome, a condition at high risk for AD type dementia [25]. It is of interest that mothers of children with Down's syndrome showed increased serum HSV-2 antibody levels [26]. Viruses of the HSV family are among the most probable pathogen candidates for brain reactivation in old age, since their possess a well known ability to escape peripheral immune responses by invading neurons. It is of interest that during aging a substantial proportion of peripheral CD8 T cytotoxic cells have been found to be directed against Epstein-Barr virus (EBV) and cytomegalovirus (CMV), which belong to the HSV family. Moreover, it has been suggested that aged immune system is no longer able to control EBV or CMV reactivation [27] and virus infection might become chronic in a large proportion of the elderly. Therefore, we speculate that with advancing age an impaired immune system might facilitate virus reactivation in the brain, especially in those subjects showing the above suggested genetic signature. Latent or chronic viral infection by CMV has been indeed found to correlate with the rate of cognitive decline in the Sacramento Area Latino Study on Aging [28]. Another study, focused on elderly with cardiovascular disease, showed that HSV and CMV burden was associated with cognitive impairment [29].

Therefore, brain infection by reactivated latent viruses might be one of the *primus movens *inducing progressive neuronal loss, astro-glia activation, and, by impairing APP transport along the axons [19], APP dis-appropriate metabolism and amyloid deposition.

This hypothesis is partially supported by data from HIV positive patients under protease inhibitor treatment and without encephalitis, where Aβ amyloid brain deposition was a common neuropathological feature [30]. Moreover it has been showed that APP, a putative receptor for the microtubule motor named kinesin, is a major component of viral HSV-1 particles, as abundant as any viral encoded protein [31].

These findings indeed showed that a brain virus infection could induce amyloid deposition. Another GWA in AD from Europe and USA recently confirmed the association of TOMM-40, PVRL-2, APOJ and APOE with AD. This investigation also signaled a significant association of the phosphatidilinositol-binding clathrin assembly protein gene (PICALM) with AD [32]. It is of interest that clathrin (CLA) mediated endocytosis is involved in internalization and transportation of viruses into the infected cell and to the nucleus. For instance, human rhinovirus is internalized by a CLA dependent mechanism [33] and adenovirus transport into motor-neuron axons is mediated via CLA endocytosis [34]. Insect parvovirus particles were also shown to be rapidly internalized into CLA-coated vesicles and slowly moved within early and late endocytic compartments to the nucleus [35]. Moreover, varicella herpes zoster virus was shown to interfere with intracellular trafficking by interacting with CLA-coated vesicles for subsequent transportation to endosomes [36]. Data from this independent GWA in AD patients also seem to support the presence of a genetic signature suggestive of a viral risk factor in AD. Finally recent data, reporting that Aβ peptide showed an anti-microbial activity [37] and acted as a defense molecule of the innate immunity, is compatible with the hypothesis of viral association with AD etiology and pathogenesis. The accumulation of Aβ and plaque deposit may derive by an over-production of Aβ peptides directed against a viral invader of the brain. Moreover, some evidence is on record showing that HSV1 can directly contribute to the processing of Aβ and to the development of senile plaques and a Ca(^2+^) dependent APP phosphorylation and Aβ 42 accumulation in rat cortical neurons [38,39].

Two recent meta-analysis from GWA [40,41] confirmed APOE, CLU, PICALM and CR-1 as susceptibility genes for AD risk. Therefore, this genetic trait in association with the other above discussed genes might represent a gene cluster affecting AD risk by influencing virus infection susceptibility. Our hypothesis describes a set of gene upstream of the APOE locus on chromosome 19 spanning from CEACAM-19 to APOE (as shown in Table 1) that may constitute a gene cluster of susceptibility for AD by affecting different mechanism involved in virus entrance or resistance to virus infection. CLU/APOJ, CR-1 and CLEC-16 genes located on different chromosome complement the AD susceptibility gene cluster also by affecting virus entry and cellular defense mechanism. It is interesting to note that SNPs upstream of APOE locus spanning from TOMM-40 to APOE promoter may also play a role in AD risk by affecting APOE expression in AD brain [42]. Moreover a genetic association study also confirmed that PVRL-2 (Nec-2), TOMM-40, APOE and APOC1 predispose to AD and showed that this region is firmly sandwiched between two recombination hotspots [17]. Therefore, the APOE ε4 might represent a genetic beacon of this set of genes located in its proximity on chromosome 19. Our hypothesis confirm and extend to other genes, a recent suggestion indicating that APP, APOE, CR-1, CLU and PICALM genes may be involved in HSV life cycle [43]. In conclusion, present findings suggest that during ageing virus reactivation may be more frequent in the elderly showing a genetic signature predisposing to an increased susceptibility for HSV and other virus infections of the brain. In these subjects the microorganisms are more likely to induce a limited, segmental and chronic sub-clinical pseudo-encephalitis resulting in progressive neurodegeneration. Further investigations will validate or refute this innovative approach to dementia in old age and clarify whether the presence of HSV and/or other infectious agents in the CNS represents a causative factor or a secondary infection in AD.

## Competing interests

The authors declare that they have no competing interests.

## Authors' contributions

EP contributed to generate part of genetic data and searched in gene bank to find biological function of candidate genes. IC searched and defined by a detailed perusal in gene bank the biological function of each gene regarding virus pathway. MI contributed to search Medline for virus association in AD. FL designed the hypothesis, supervised gene bank and medline data mining and wrote most of the paper. All authors read and approved the final manuscript.
